# A stratified adaptive two-stage design with co-primary endpoints for phase II clinical oncology trials

**DOI:** 10.1186/s12874-022-01748-w

**Published:** 2022-10-26

**Authors:** Bastien Cabarrou, Eve Leconte, Patrick Sfumato, Jean-Marie Boher, Thomas Filleron

**Affiliations:** 1 Biostatistics & Health Data Science Unit, Institut Claudius Regaud - IUCT-O, 1 avenue Irène Joliot-Curie, 31059 Cedex 9 Toulouse, France; 2 Toulouse School of Economics, University of Toulouse Capitole, Toulouse, France; 3 Biostatistics Unit, Institut Paoli-Calmettes, Marseille, France; 4 Aix Marseille Université, INSERM, IRD, SESSTIM, Marseille, France

**Keywords:** Phase II clinical oncology trials, Heterogeneity, Adaptive stratified design, Co-primary endpoints

## Abstract

**Background:**

Given the inherent challenges of conducting randomized phase III trials in older cancer patients, single-arm phase II trials which assess the feasibility of a treatment that has already been shown to be effective in a younger population may provide a compelling alternative. Such an approach would need to evaluate treatment feasibility based on a composite endpoint that combines multiple clinical dimensions and to stratify older patients as fit or frail to account for the heterogeneity of the study population to recommend an appropriate treatment approach. In this context, stratified adaptive two-stage designs for binary or composite endpoints, initially developed for biomarker studies, allow to include two subgroups whilst maintaining competitive statistical performances. In practice, heterogeneity may indeed affect more than one dimension and incorporating co-primary endpoints, which independently assess each individual clinical dimension, would therefore appear quite pertinent. The current paper presents a novel phase II design for co-primary endpoints which takes into account the heterogeneity of a population.

**Methods:**

We developed a stratified adaptive Bryant & Day design based on the Jones et al. and Parashar et al. algorithm. This two-stage design allows to jointly assess two dimensions (e.g. activity and toxicity) in two different subgroups. The operating characteristics of this new design were evaluated using examples and simulation comparisons with the Bryant & Day design in the context where the study population is stratified according to a pre-defined criterion.

**Results:**

Simulation results demonstrated that the new design minimized the expected and maximum sample sizes as compared to parallel Bryant & Day designs (one in each subgroup), whilst controlling type I error rates and maintaining a competitive statistical power as well as a high probability of detecting heterogeneity.

**Conclusions:**

In a heterogeneous population, this two-stage stratified adaptive phase II design provides a useful alternative to classical one and allows to identify a subgroup of interest without dramatically increasing sample size. As heterogeneity is not limited to older populations, this new design may also be relevant to other study populations such as children or adolescents and young adults or the development of targeted therapies based on a biomarker.

**Supplementary Information:**

The online version contains supplementary material available at 10.1186/s12874-022-01748-w.

## Background

The main objective of a phase II oncology trial is to assess the anti-tumoral activity of an experimental treatment. If promising results are obtained, the phase II is followed by a phase III trial to evaluate the effectiveness of an experimental treatment compared to a standard treatment. Older patients are vastly underrepresented in phase III clinical trials and the problem of recruiting older people has been largely documented in the literature. The most common barriers cited were: stringent eligibility criteria, oncologists concerns for toxicity, patients and family refusal [1]. Given the challenges of conducting randomized phase III trials in older patients, several authors have previously suggested conducting single-arm phase II trials to assess the feasibility of a treatment that has been shown to be effective in a younger population [2, 3]. Indeed, perhaps more importantly than in any other population, cancer care should not compromise quality of life or autonomy [2, 3]. Treatment feasibility can be evaluated with a composite endpoint combining multiple clinical dimensions (e.g. activity, toxicity, quality of life, etc.). The treatment may be considered feasible if it fulfills some or all components of the composite endpoint. Another conundrum is to take into account the heterogeneity of this population and stratifying older patients as fit or frail is crucial to recommend an appropriate treatment approach [4]. Classical phase II designs for binary or composite endpoints [5–7] do not deal with this heterogeneity and can lead to erroneous conclusions in an unselected population, while a specific subgroup of less frail (or less fit) patients might benefit (or not) from the new therapeutic. Stratified adaptive two-stage designs for binary or composite endpoints, which allow the inclusion of two subgroups and identify one of interest at the end of the first or the second stage, have recently been proposed [8–10]. Initially developed for biomarker studies, these types of approaches can also be applied to geriatric clinical oncology trials and allow to minimize the sample size whilst maintaining a competitive statistical performance that is comparable to classical approaches [11]. These stratified adaptive designs have been developed for binary or composite endpoints and they take into account the heterogeneity of a population when considering a single or combined clinical dimensions where each of them theoretically carries the same clinical importance. However, depending on the clinical context, the impact on autonomy or quality of life may take precedence over anti-tumoral activity in treatment decision-making. Moreover, interpretation may be difficult if there are divergent results for each clinical dimension separate. Thus, the use of co-primary endpoints that assess each clinical dimension independently appears more relevant in this light [12]. Several designs that deal with these types of endpoints have been proposed, but the most widely used is the one developed by Bryant and Day [13]. To the best of our knowledge the current literature does not include any reports of phase II designs for co-primary endpoints that account for heterogeneity. The current paper therefore details a stratified adaptive Bryant & Day (SABD) design based on the algorithm developed by Jones et al. [8] and Parashar et al. [10] (Methods section). The operating characteristics of the novel design are then evaluated using examples and simulation comparisons with the Bryant & Day (BD) design (Results section).

## Methods

### Bryant & Day (BD) design

**Fig. 1:**
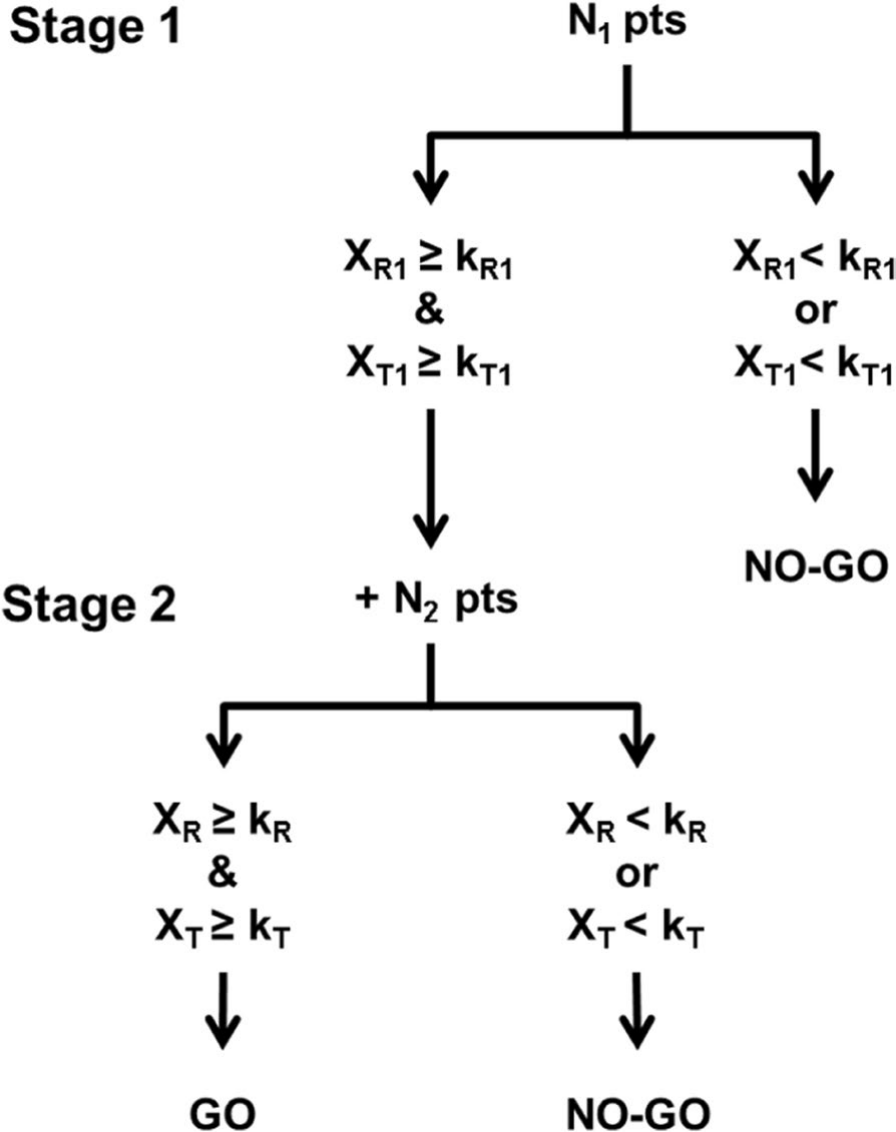
Bryant & Day (BD) design

The BD design can be considered as a two-stage Simon optimal design [6] which considers two dimensions as co-primary endpoints, namely activity and toxicity. The BD design, where *X*_*R*1_ and *X*_*T*1_ represent the number of responses and non-toxicities observed at the end of the first stage and *X*_*R*_ and *X*_*T*_ the total number of responses and non-toxicities observed at the end of the second stage, is shown in Fig. 1.

After the inclusion of *N*_1_ patients, the study will be stopped for futility if an insufficient number of responses or non-toxicities are observed (i.e. *X*_*R*1_ < *k*_*R*1_ or *X*_*T*1_ < *k*_*T*1_). The experimental treatment will be considered as promising (i.e. «go-decision») if a sufficient number of responses and non-toxicities are observed in the interim (i.e. *X*_*R*1_ ≥ *k*_*R*1_ and *X*_*T*1_ ≥ *k*_*T*1_) and in the final (i.e. *X*_*R*_ ≥ *k*_*R*_ and *X*_*T*_ ≥ *k*_*T*_) analysis.

Unacceptable and acceptable rates for each dimension are denoted as follows, with *p*_*R*_ and *p*_*T*_ respectively representing the response rate and the non-toxicity rate:

- *p*_*R*0_: unacceptable response rate
- *p*_*R*1_: acceptable response rate
- *p*_*T*0_: unacceptable non-toxicity rate
- *p*_*T*1_: acceptable non-toxicity rate

Given the two-dimensional nature of the endpoint, the null and alternative hypotheses are areas and defined by *H*_0_: {*p*_*R*_ ≤ *p*_*R*0_ or *p*_*T*_ ≤ *p*_*T*0_} and *H*_1_: {*p*_*R*_ > *p*_*R*0_ and *p*_*T*_ > *p*_*T*0_}, respectively. Four particular hypotheses corresponding to four possible states are considered:

- *H*_00_: {*p*_*R*_ = *p*_*R*0_ and *p*_*T*_ = *p*_*T*0_}
- *H*_01_: {*p*_*R*_ = *p*_*R*0_ and *p*_*T*_ = *p*_*T*1_}
- *H*_10_: {*p*_*R*_ = *p*_*R*1_ and *p*_*T*_ = *p*_*T*0_}
- *H*_11_: {*p*_*R*_ = *p*_*R*1_ and *p*_*T*_ = *p*_*T*1_}

There are four associated error rates:

- *α:* is the probability of considering the treatment as promising in the case where true response and non-toxicity rates are considered as unacceptable (i.e. under *H*_00_),
- *α*_*R*_: is the probability of considering the treatment as promising in the case where true response and non-toxicity rates are considered as unacceptable and acceptable, respectively (i.e. under *H*_01_),
- *α*_*T*_: is the probability of considering the treatment as promising in the case where true response and non-toxicity rates are considered as acceptable and unacceptable, respectively (i.e. under *H*_10_),
- *β*: is the probability of considering the treatment as insufficiently promising in the case where true response and non-toxicity rates are considered as acceptable (i.e. under *H*_11_).

Sample sizes of stage 1 and 2 (*N*_1_ and *N*_2_) and stopping boundaries (*k*_*R*1_, *k*_*T*1_, *k*_*R*_ and *k*_*T*_) are determined from the specified values for *p*_*R0*_, *p*_*T0*_, *p*_*R1*_ and *p*_*T1*_ and the type I (*α*_*R*_ and *α*_*T*_) and type II (*β*) error rates. The optimal design is defined as the one that minimizes the maximum expected sample size (ESS) under *H*_10_ or *H*_01_ (i.e. max{ESS under *H*_10_, ESS under *H*_01_}) whilst controlling for *α*_*R*_, *α*_*T*_ and *β*.

### Stratified Adaptive Bryant & Day (SABD) design

**Fig. 2:**
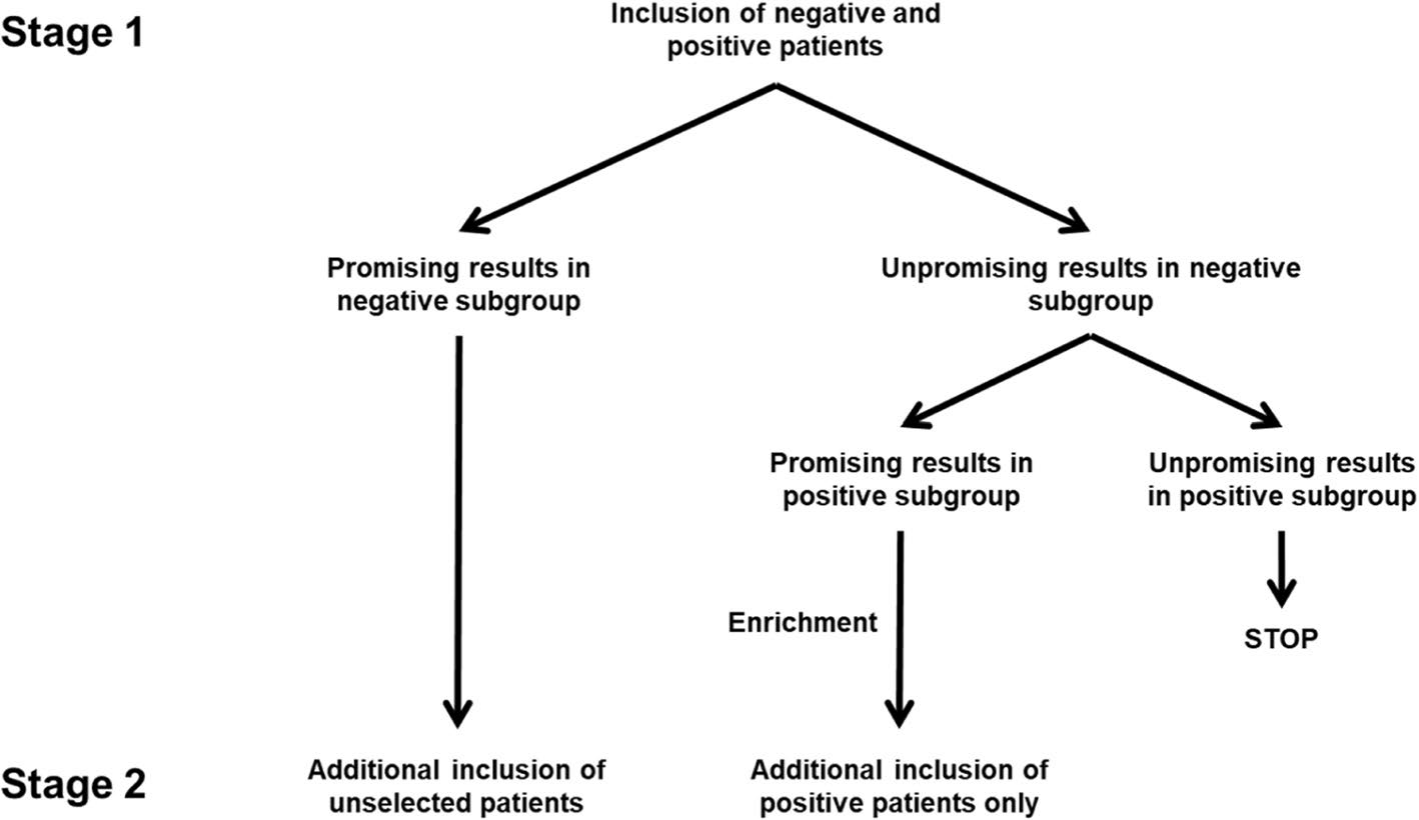
Jones et al. and Parashar et al. algorithm

To take into account population heterogeneity, we developed a SABD design based on the Jones et al. [8] and Parashar et al. [10] algorithm. As compared to these designs that have been developed for binary or composite endpoints, this novel two-stage design allows to jointly assess two clinical dimensions (e.g. activity and toxicity) through co-primary endpoints in two different subgroups and to identify one of interest at the end of the first or the second stage. In the context of a geriatric clinical oncology trial for example, this allows patients to be stratified, according to a geriatric criterion, into frail and fit subgroups. To simplify the notation, these two subgroups will be defined as negative (‹‹-››) and positive (‹‹ + ››) subgroups respectively. The two-stage algorithm proposed by Jones et al. and Parashar et al., presented in Fig. 2, relies on an assumption of hierarchy between the subgroups as the true response and non-toxicity rates will always be equal or higher in the positive subgroup than in the negative subgroup. This implies that, according to the preliminary results observed at the end of the first stage, enrollment continues in an unselected population if promising results are observed in the negative subgroup, or in the positive subgroup (i.e. enrichment) if promising results are observed in this subgroup only.

**Fig. 3:**
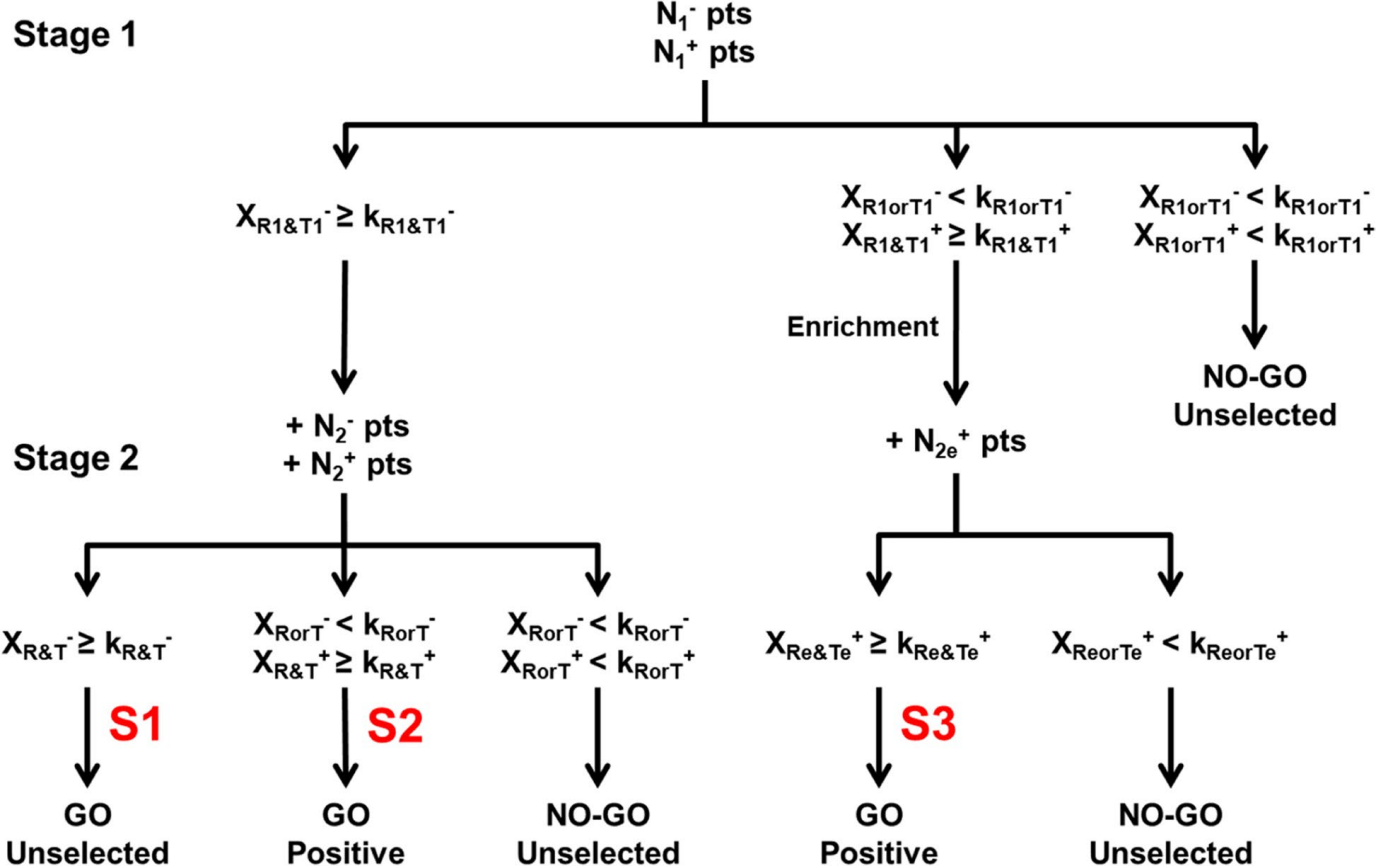
Stratified Adaptive Bryant & Day (SABD) design. *e*: **‹‹**enrichment**››**. + : **‹‹**inclusion of additional**››**. *N*_1_^−^ and *N*_1_^+^: number of patients to be included at the first stage. *N*_2_^−^, *N*_2_^+^ and *N*_2e_^+^: number of patients to be included at the second stage. *N*^−^ = *N*_1_^−^ + *N*_2_^−^, *N*^+^ = *N*_1_^+^ + *N*_2_^+^ and *N*_e_^+^ = *N*_1_^+^ + *N*_2e_^+^: total number of patients to be included. *k*_R1_^−^, *k*_T1_^−^, *k*_R1_^+^ and *k*_T1_^+^: first stage stopping boundaries. *k*_R_^−^, *k*_T_^−^, *k*_R_^+^, *k*_T_^+^, *k*_Re_^+^ and *k*_Te_^+^: second stage stopping boundaries. *X*_R1_^−^, *X*_T1_^−^, *X*_R1_^+^ and *X*_T1_^+^: number of responses and non-toxicities observed during the first stage. *X*_R2_^−^, *X*_T2_^−^ *X*_R2_^+^, *X*_T2_^+^, *X*_R2e_^+^ and *X*_T2e_^+^: number of responses and non-toxicities observed during the second stage. *X*_R_^−^ = *X*_R1_^−^ + *X*_R2_^−^, *X*_T_^−^ = *X*_T1_^−^ + *X*_T2_^−^, *X*_R_^+^ = *X*_R1_^+^ + *X*_R2_^+^, *X*_T_^+^ = *X*_T1_^+^ + *X*_T2_^+^, *X*_Re_^+^ = *X*_R1_^+^ + *X*_R2e_^+^ and *X*_Te_^+^ = *X*_T1_^+^ + *X*_T2e_^+^: total number of responses and non-toxicities observed

Based on this algorithm and adapted from the BD design to consider two co-primary endpoints, we proposed the SABD design presented in Fig. 3.

The study begins with the inclusion of *N*_1_^−^ and *N*_1_^+^ patients in the negative and positive subgroup, respectively. According to the results observed at the end of the first stage, enrollment will be stopped for futility if an insufficient number of responses or non-toxicities are observed in the two subgroups (i.e. (*X*_*R*1_^−^ < *k*_*R*1_^−^ or *X*_*T*1_^−^ < *k*_*T*1_^−^) and (*X*_*R*1_^+^  < *k*_*R*1_^+^ or *X*_*T*1_^+^  < *k*_*T*1_^+^)). Otherwise, enrollment will continue in the unselected population if a sufficient number of responses and non-toxicities are observed in the negative subgroup (i.e. *X*_*R*1_^−^ ≥ *k*_*R*1_^−^ and *X*_*T*1_^−^ ≥ *k*_*T*1_^−^). If a sufficient number of responses and non-toxicities are only observed in the positive subgroup (i.e. (*X*_*R*1_^−^ < *k*_*R*1_^−^ or *X*_*T*1_^−^ < *k*_*T*1_^−^) and (*X*_*R*1_^+^  ≥ *k*_*R*1_^+^ and *X*_*T*1_^+^  ≥ *k*_*T*1_^+^)) then enrollment will continue in this subgroup only. At the end of the second stage, the experimental treatment may be considered as promising (i.e. ‹‹go-decision››) in the two subgroups (i.e. S1) or in the positive subgroup only (i.e. S2 or S3).

#### Hypotheses

Similarly to the BD design, our SABD design assumes that the co-primary endpoints are independent in the two subgroups. If *p*_*R*_^−^, *p*_*R*_^+^, *p*_*T*_^−^ and *p*_*T*_^+^ respectively correspond to the true response and non-toxicity rates in the negative and positive subgroups, the unacceptable and acceptable rates for each endpoint and subgroup may then be expressed as follows:

- *p*_*R*0_^−^ and *p*_*R*0_^+^: unacceptable response rates in the negative and positive subgroups,
- *p*_*R*1_^−^ and *p*_*R*1_^+^: acceptable response rates in the negative and positive subgroups,
- *p*_*T*0_^−^ and *p*_*T*0_^+^: unacceptable non-toxicity rates in the negative and positive subgroups,
- *p*_*T*1_^−^ and *p*_*T*1_^+^: acceptable non-toxicity rates in the negative and positive subgroups.

It is assumed that the null hypothesis is identical between subgroups for the co-primary endpoints (i.e. *p*_*R*0_^−^ = *p*_*R*0_^+^ and *p*_*T*0_^−^ = *p*_*T*0_^+^). Null and alternative hypotheses in both subgroups are therefore defined as follows:

- *H*_0_^−(+)^: {*p*_*R*_^−(+)^ ≤ *p*_*R*0_^−(+)^ or *p*_*T*_^−(+)^ ≤ *p*_*T*0_^−(+)^}
- *H*_1_^−(+)^: {*p*_*R*_^−(+)^ > *p*_*R*0_^−(+)^ and *p*_*T*_^−(+)^ > *p*_*T*0_^−(+)^}

Four particular hypotheses in both subgroups are considered:

- *H*_00_^−(+)^: {*p*_*R*_^−(+)^ = *p*_*R*0_^−(+)^ and *p*_*T*_^−(+)^ = *p*_*T*0_^−(+)^}
- *H*_10_^−(+)^: {*p*_*R*_^−(+)^ = *p*_*R*1_^−(+)^ and *p*_*T*_^−(+)^ = *p*_*T*0_^−(+)^}
- *H*_01_^−(+)^: {*p*_*R*_^−(+)^ = *p*_*R*0_^−(+)^ and *p*_*T*_^−(+)^ = *p*_*T*1_^−(+)^}
- *H*_11_^−(+)^: {*p*_*R*_^−(+)^ = *p*_*R*1_^−(+)^ and *p*_*T*_^−(+)^ = *p*_*T*1_^−(+)^}

#### Probability of rejecting null hypotheses

There are three possible scenarios where the experimental treatment is considered as promising in the unselected population or only in the positive subgroup (i.e. ‹‹go-decision››). These scenarios correspond to S1, S2 and S3 presented in Fig. 3.

The probability of considering the experimental treatment as promising in the unselected population (i.e. reject *H*_0_^−^ and *H*_0_^+^) according to S1 is defined as:

$$P\left(S1\vert p_R^-,p_T^-\right)=P(X_{R1}^-+X_{R2}^-\geq k_{R\;}^-and\;X_{R1}^-\geq k_{R1}^-)\times P(X_{T1}^-+X_{T2}^-\geq k_T^-\;and\;X_{T1}^-\geq k_{T1}^-)$$

According to the hypothesis of hierarchy between subgroups, the probability of considering the experimental treatment as promising depends on the true response and non-toxicity rate in the negative subgroup only.

The probability of considering the experimental treatment as promising in the positive subgroup only (i.e. reject *H*_0_^+^) according to S2 is defined as:

$$P\left(S2\left|p_R^-,\;\right.p_T^-,\;p_R^+,\;p_T^+\right)=P\left(X_R^+\geq k_R^+\right)\times P\left(X_T^+\geq k_T^+\right)\times\left[P\left(\left(X_{R1}^-+X_{R2}^-<k_R^-\;and\;X_{R1}^-\geq k_{R1}^-\;and\;X_{T1}^-\geq k_{T1}^-\right)\;or\;\left(X_{T1}^-\;+\;X_{T2}^-<k_T^-\;and\;X_{R1}^-\geq k_{R1}^-\;and\;X_{T1}^-\geq k_{T1}^-\right)\right)\right]$$

The probability of considering the experimental treatment as promising in the positive subgroup only (i.e. reject *H*_0_^+^) according to S3 is defined as:

$$P\left(S3\vert p_R^-,p_T^-,p_R^+,p_T^+\right)=P\left(X_{R1}^++X_{R2e}^+\geq k_{Re}^+\;and\;X_{R1}^+\geq k_{R1}^+\right)\times P\left(X_{T1}^++X_{T2e}^+\geq k_{Te}^+\;and\;X_{T1}^+\geq k_{T1}^+\right)\times P(X_{R1}^-<k_{R1}^-\;or\;X_{T1}^-<k_{T1}^-)$$

To compute probabilities of rejecting null hypotheses, it is assumed that the number of responses and non-toxicities follow a binomial distribution, *B*(*N*,*p*), with parameters *N* and *p* defined in Table 1.

**Table 1:** Parameters of binomial distributions

	Response	Non-toxicity
Negative	*X*_*R*1_*-~B*(*N*_1_*-,p*_*R*_-)	*X*_*T*__1_^-^~*B*(*N*_1_^-^,*p*_*T*_^-^)
	*X*_*R*__2_^-^~*B*(*N*_2_^-^,*p*_*R*_^-^)	*X*_*T*__2_^-^~*B*(*N*_2_^-^,*p*_*T*_^-^)
Positive	*X*_*R*__1_^+^~*B*(*N*_1_^+^,*p*_*R*_^+^)	*X*_*T*__1_^+^~*B*(*N*_1_^+^,*p*_*T*_^+^)
	*X*_*R*_^+^~*B*(*N*^+^,*p*_*R*_^+^)	*X*_*T*_^+^~*B*(*N*^+^,*p*_*T*_^+^)
	*X*_*R2e*_^+^~*B*(*N*_*2e*_^+^,*p*_*R*_^+^)	*X*_*T2e*_^+^~*B*(*N*_*2e*_^+^,*p*_*T*_^+^)

#### Type I errors

Similarly to the BD design, three type I errors may be considered for the SABD design. The overall type I error rate *α* corresponds to the probability of considering the treatment as promising in the unselected population or in the positive subgroup in the case where true response and non-toxicity rates are considered as unacceptable in the two subgroups (i.e. under *H*_00_^−^ and *H*_00_^+^). It is defined as:

$$\alpha =P\left(S1|{p}_{R0}^{-},{p}_{T0}^{-}\right)+P\left(S2|{p}_{R0}^{-},{p}_{T0}^{-},{p}_{R0}^{+},{p}_{T0}^{+}\right)+P\left(S3|{p}_{R0}^{-},{p}_{T0}^{-},{p}_{R0}^{+},{p}_{T0}^{+}\right)$$

Type I error rate *α*_*R*_ corresponds to the probability of considering the treatment as promising in the unselected population or in the positive subgroup in the case where true response and non-toxicity rates are considered as unacceptable and acceptable, respectively, in the two subgroups (i.e. under *H*_01_^−^ and *H*_01_^+^). It is defined as:

$${\alpha }_{R}=P\left(S1|{p}_{R0}^{-},{p}_{T1}^{-}\right)+P\left(S2|{p}_{R0}^{-},{p}_{T1}^{-},{p}_{R0}^{+},{p}_{T1}^{+}\right)+P\left(S3|{p}_{R0}^{-},{p}_{T1}^{-},{p}_{R0}^{+},{p}_{T1}^{+}\right)$$

Type I error rate *α*_*T*_ corresponds to the probability of considering the treatment as promising in the unselected population or in the positive subgroup in the case where true response and non-toxicity rates are considered as acceptable and unacceptable, respectively, in the two subgroups (i.e. under *H*_10_^−^ and *H*_10_^+^). It is defined as:

$${\alpha }_{T}=P\left(S1|{p}_{R1}^{-},{p}_{T0}^{-}\right)+P\left(S2|{p}_{R1}^{-},{p}_{T0}^{-},{p}_{R1}^{+},{p}_{T0}^{+}\right)+P\left(S3|{p}_{R1}^{-},{p}_{T0}^{-},{p}_{R1}^{+},{p}_{T0}^{+}\right)$$

#### Statistical power

The probability of considering the treatment as promising in the unselected population in the case where true response and non-toxicity rates are considered as acceptable in the negative subgroup, and therefore in the positive subgroup by the assumption of hierarchy, corresponds to *P*(*S*1|*p*_*R1*_^−^,*p*_*T1*_^−^). The probability of considering the treatment as promising in the positive subgroup in the case where true response and non-toxicity rates are considered as acceptable in the positive subgroup only corresponds to *P*(S2|*p*_*R0*_^−^,*p*_*T0*_^−^,*p*_*R1*_^+^,*p*_*T1*_^+^) + *P*(*S*3|*p*_*R0*_^−^,*p*_*T0*_^−^,*p*_*R1*_^+^,*p*_*T1*_^+^). As proposed by Parashar et al. [10], the overall power is defined by the minimum of these two probabilities:

$$power=1-\beta =\mathrm{min}\{P\left(S1|{p}_{R1}^{-},{p}_{T1}^{-}\right),P\left(S2|{p}_{R0}^{-},{p}_{T0}^{-},{p}_{R1}^{+},{p}_{T1}^{+}\right)+P\left(S3|{p}_{R0}^{-},{p}_{T0}^{-},{p}_{R1}^{+},{p}_{T1}^{+}\right)\}$$

#### Expected sample size (ESS) and optimal design

A minimum of *N*_1_^−^ + *N*_1_^+^ patients need to be included. According to the number of responses and non-toxicities observed in the interim analysis, three scenarios are considered: none or *N*_2_^−^ + *N*_2_^+^ or *N*_2e_^+^ additional patients will need to be included at the second stage. The ESS is determined as follows:

$$ESS\left({p}_{R}^{-},{p}_{T}^{-},{p}_{R}^{+},{p}_{T}^{+}\right)={N}_{1}^{-}+{N}_{1}^{+}+\left({N}_{2}^{-}+{N}_{2}^{+}\right)\times P\left({X}_{R1}^{-}\ge {k}_{R1}^{-}\right)\times P\left({X}_{T1}^{-}\ge {k}_{T1}^{-}\right)+{N}_{2e}^{+}\times P\left({X}_{R1}^{+}\ge {k}_{R1}^{+}\right)\times P\left({X}_{T1}^{+}\ge {k}_{T1}^{+}\right)\times P\left({X}_{R1}^{-}<{k}_{R1}^{-} \;or\; {X}_{T1}^{-}<{k}_{T1}^{-}\right)$$

As proposed by Parashar et al. [10], the optimal design (*k*_R1_^−^, *k*_T1_^−^, *k*_R1_^+^, *k*_T1_^+^, *N*_1_^−^, *N*_1_^+^, *k*_Re_^+^, *k*_Te_^+^, *N*_e_^+^, *k*_R_^−^, *k*_T_^−^, *k*_R_^+^, *k*_T_^+^, *N*^−^, *N*^+^) is defined as the one that minimizes the maximum ESS under (*H*_01_^−^,*H*_01_^+^) or (*H*_10_^−^,*H*_10_^+^) (i.e. $\mathrm{max}\{ESS\left({p}_{R0}^{-},{p}_{T1}^{-},{p}_{R0}^{+},{p}_{T1}^{+}\right),ESS\left({p}_{R1}^{-},{p}_{T0}^{-},{p}_{R1}^{+},{p}_{T0}^{+}\right)\}$) while controlling type I (*α*_*R*_ and *α*_*T*_) and type II (*β*) error rates. To determine the optimal design, 15 parameters need to be estimated. To reduce the computational burden, a similar approach to the one proposed by Jones et al. [8] is used. Parameters (*N*_1_^−^, *N*^−^, *k*_R1_^−^, *k*_T1_^−^, *k*_R_^−^, *k*_T_^−^) and (*N*_1_^+^, *k*_R1_^+^, *k*_T1_^+^) are derived from the BD design with (*p*_*R0*_^−^, *p*_*T0*_^−^, *p*_*R1*_^−^, *p*_*T1*_^−^, *α*_*R*_/2, *α*_*T*_/2, *β*) and (*p*_*R0*_^+^, *p*_*T0*_^+^, *p*_*R1*_^+^, *p*_*T1*_^+^, *α*_*R*_/2, *α*_*T*_/2, *β*), respectively (type I error rates are set at *α*_*R*_/2 and *α*_*T*_/2 to adjust for multiplicity). To delineate the parameter search space, the maximum sample size is set at 2 × *N*^−^.

#### Probability of Early termination (PET)

The study will stop for futility if an insufficient number of responses or non-toxicities are observed in both groups in the interim analysis. The PET is determined as follows:

$$PET\left({p}_{R}^{-},{p}_{T}^{-},{p}_{R}^{+},{p}_{T}^{+}\right)=P\left({X}_{R1}^{-}<{k}_{R1}^{-} \;or\; {X}_{T1}^{-}<{k}_{T1}^{-}\right)\times P\left({X}_{R1}^{+}<{k}_{R1}^{+} \;or\; {X}_{T1}^{+}<{k}_{T1}^{+}\right)$$

## Results

### Examples of SABD design

Three examples of the SABD design are considered. In the first example, hypotheses are based on the GERICO10 phase II trial which aimed to evaluate the feasibility of a chemotherapy treatment with docetaxel-prednisone in patients age 75 or older, classified as vulnerable or frail according to the International Society of Geriatric Oncology criteria, with castration-resistant metastatic prostate cancer [14]. Same hypotheses are defined for the two co-primary endpoints in the two subgroups (*p*_*R*0_^−(+)^ = *p*_*T*0_^−(+)^ = 0.70 and *p*_*R*1_^−(+)^ = *p*_*T*1_^−(+)^ = 0.90). In the second example, different hypotheses are defined between the two co-primary endpoints in the two subgroups (*p*_*R*0_^−(+)^ = 0.30, *p*_*T*0_^−(+)^ = 0.60, *p*_*R*1_^−(+)^ = 0.60 and *p*_*T*1_^−(+)^ = 0.90). In the third example, different hypotheses are defined between the two co-primary endpoints and between the two subgroups for non-toxicity (*p*_*R*0_^−(+)^ = 0.10, *p*_*T*0_^−(+)^ = 0.60, *p*_*R*1_^−(+)^ = 0.40, *p*_*T*1_^−^ = 0.80 and *p*_*T*1_^+^  = 0.90). Type I error rates (*α*_*R*_ and *α*_*T*_) and overall power (1-*β*) are set at 10% and 80%, respectively. The hypotheses, parameters and operating characteristics for the three examples are summarized in Table 2.

**Table 2:** Examples of stratified adaptive Bryant & Day (SABD) design (*ESS*_RiTj_ and PET_RiTj_ correspond to *ESS* (*p*_*Ri*_^−^,*p*_*Tj*_^−^,*p*_*Ri*_^+^,*p*_*Tj*_^+^) and *PET* (*p*_*Ri*_^−^,*p*_*Tj*_^−^,*p*_*Ri*_^+^,*p*_*Tj*_^+^), respectively)

Hypotheses				Parameters								Operating characteristics		
*p*_*R*0_	*p*_*R*1_^−^ *p*_*R*1_^+^	*p*_*T*0_	*p*_*T*1_^−^ *p*_*T*1_^+^	*k*_R1_^−^ *k*_R1_^+^	*k*_T1_^−^ *k*_T1_^+^	*N*_1_^−^ *N*_1_^+^	*k*_Re_^+^ *k*_Te_^+^	*N*_e_^+^	*k*_R_^−^ *k*_R_^+^	*k*_T_^−^ *k*_T_^+^	*N*^−^ *N*^+^	Attained *α*_*R*_ / *α*_*T*_ / 1- *β*	max (*ESS*_*R*0T1_,*ESS*_*R*1T0_)	min (*PET*_R0T1,_*PET*_R1T0_)
0.70	0.90 0.90	0.70	0.90 0.90	8 8	8 8	10 10	29 29	35	29 27	29 27	35 32	0.094 / 0.094 / 0.800	42.5	0.415
0.30	0.60 0.60	0.60	0.90 0.90	4 4	7 7	9 9	10 16	21	11 8	18 13	23 16	0.094 / 0.093 / 0.800	25.7	0.554
0.10	0.40 0.40	0.60	0.80 0.90	3 2	12 7	17 9	4 13	16	7 3	26 9	35 10	0.087 / 0.096 / 0.800	32.1	0.580

In the first example, a maximum of 67 patients need to be included and the interim analysis is performed after the enrollment of 10 patients into each subgroup. According to the number of responses and non-toxicities observed at the end of the first stage, three scenarios are possible: the study is stopped for futility if at most 7 responses or non-toxicities are observed in the negative and positive subgroups; enrollment continues in an unselected population with the recruitment of additional 25 (*N*_*2*_^−^ = *N*^−^-*N*_1_^−^) and 22 (*N*_*2*_^+^  = *N*^+^-*N*_1_^+^) patients in the negative and positive subgroups, respectively, if at least 8 responses and non-toxicites are observed in the negative subgroup; enrollment continues in the positive subgroup only with the recruitment of additional 25 patients (enrichment: *N*_*2e*_^+^  = *N*_e_^+^-*N*_1_^+^) if at most 7 responses and non-toxicities are observed in the negative subgroup and at least 8 responses and non-toxicites are observed in the positive subgroup. At the end of the second stage after the enrollment of 35 and 32 patients in the negative and positive subgroups, respectively, a «go-decision» is declared in the unselected population or in the positive subgroup only if at least 29 responses and 27 non-toxicites are observed in the negative or in the positive subgroup only, respectively. After the enrollment of 35 patients in the positive subgroup (enrichment), a «go-decision» is declared in the positive subgroup only if at least 29 responses and 29 non-toxicites are observed. The ESS and the PET for insufficient activity and/or excessive toxicity equate to 42.5 patients and 41.5%, respectively.

In the second example, a maximum of 39 patients need to be included and the interim analysis is performed after the enrollment of 9 patients into each subgroup. According to the number of responses and non-toxicities observed at the end of the first stage, three scenarios are possible: the study is stopped for futility; enrollment continues in an unselected population with the recruitment of additional 14 (*N*_*2*_^−^ = *N*^−^-*N*_1_^−^) and 7 (*N*_*2*_^+^  = *N*^+^-*N*_1_^+^) patients in the negative and positive subgroups, respectively; enrollment continues in the positive subgroup only with the recruitment of additional 12 patients (enrichment: *N*_*2e*_^+^  = *N*_e_^+^-*N*_1_^+^). The ESS and the PET for insufficient activity and/or excessive toxicity equate to 25.7 patients and 55.4%, respectively.

In the third example, a maximum of 45 patients need to be included and the interim analysis is performed after the enrollment of 17 and 9 patients in the negative and positive subgroups, respectively. According to the number of responses and non-toxicities observed at the end of the first stage, three scenarios are possible: the study is stopped for futility; enrollment continues in an unselected population with the recruitment of additional 18 (*N*_*2*_^−^ = *N*^−^-*N*_1_^−^) and 1 (*N*_*2*_^+^  = *N*^+^-*N*_1_^+^) patients in the negative and positive subgroups, respectively; enrollment continues in the positive subgroup only with the recruitment of additional 7 patients (enrichment: *N*_*2e*_^+^  = *N*_e_^+^-*N*_1_^+^). The ESS and the PET for insufficient activity and/or excessive toxicity equate to 32.1 patients and 58.0%, respectively.

A selection of SABD designs with pre-specified hypotheses are detailed in Supplementary Table 1.

An optimal SABD design requires a total of 15 parameters to be estimated. This involves a very large number of combinations and therefore necessitates an extensive computational effort when using standard software. For example, the computation time needed to determine an optimal SABD design can vary from a few minutes or hours to several weeks, depending on the hypotheses, using R software (https://cran.r-project.org/).

### Simulation studies

Simulations were carried out to investigate the operating characteristics of the SABD design and to compare to a parallel BD design (i.e. two parallel studies with one BD design in each subgroup). Three case studies corresponding to the three examples presented in previous section were considered. Type I error rate and power, for the SABD design, were set at 10% and 80%, respectively. In the parallel BD design, adjustment for multiplicity was performed to achieve an adequate overall type I error rate and sufficient statistical power to draw meaningful conclusions in the unselected population or only in the positive subgroup. Type I error rate and power were therefore set at 5% (i.e. *α*_*R*_/2 and *α*_*T*_/2) and 90% (i.e. 1—*β*/2) in each subgroup for parallel BD design, respectively. Four scenarios were considered:

- Scenario 1A: simulations were performed under *H*_01_^−(+)^ (*p*_*R*_^−(+)^ = *p*_*R*0_^−(+)^ and *p*_*T*_^−(+)^ = *p*_*T*1_^−(+)^) to assess type I error rate *α*_*R*_ and PET.
- Scenario 1B: simulations were performed under *H*_10_^−(+)^ (*p*_*R*_^−(+)^ = *p*_*R*1_^−(+)^ and *p*_*T*_^−(+)^ = *p*_*T*0_^−(+)^) to assess type I error rate *α*_*T*_ and PET.
- Scenario 2: simulations were performed under *H*_00_^−^ (*p*_*R*_^−^ = *p*_*R*0_^−^ and *p*_*T*_^−^ = *p*_*T*0_^−^) and *H*_11_^+^ (*p*_*R*_^+^  = *p*_*R*1_^+^ and *p*_*T*_^+^  = *p*_*T*1_^+^) to evaluate the probability of detecting heterogeneity at the first stage (i.e. stop enrollment for futility in the negative subgroup) and the probability of considering the treatment as promising in the positive subgroup (i.e. reject *H*_0_^+^).
- Scenario 3: simulations were performed under *H*_11_^−(+)^ (*p*_*R*_^−(+)^ = *p*_*R*1_^−(+)^ and *p*_*T*_^−(+)^ = *p*_*T*1_^−(+)^) to evaluate the probability of considering the treatment as promising in the unselected population (i.e. reject *H*_0_^−^ and *H*_0_^+^).

For each case study and scenario, 100 000 hypothetical trials were simulated. The number of responses and non-toxicities were randomly generated using binomial distributions *B*(*N*,*p*) with *N* corresponding to the number of patients presented in Table 2 (*N*_1_^−^, *N*_1_^+^, *N*^−^—*N*_1_^−^, *N*^+^—*N*_1_^+^ and *N*_e_^+^—*N*_1_^+^) and *p* corresponding to the true response and non-toxicity rates defined above (*p*_*R*_^−^, *p*_*T*_^−^, *p*_*R*_^+^ and *p*_*T*_^+^).

The ESSs were also estimated for each case study and scenario. Simulation results are presented in Table 3.

**Table 3:** Simulation results

			Parallel BD	SABD
**Case study 1**				
		Maximum sample size	88	67
1A	*p*_*R*_^−^ = 0.7/*p*_*T*_^−^ = 0.9 *p*_*R*_^+^ = 0.7/*p*_*T*_^+^ = 0.9	Expected sample size	52.8	42.4
		Probability of early termination	0.458	0.416
		Probability of rejecting *H*_0_^−^ or *H*_0_^+^	0.092	0.094
1B	*p*_*R*_^−^ = 0.9/*p*_*T*_^−^ = 0.7 *p*_*R*_^+^ = 0.9/*p*_*T*_^+^ = 0.7	Expected sample size	52.8	42.4
		Probability of early termination	0.457	0.416
		Probability of rejecting *H*_0_^−^ or *H*_0_^+^	0.092	0.094
2	*p*_*R*_^−^ = 0.7/*p*_*T*_^−^ = 0.7 *p*_*R*_^+^ = 0.9/*p*_*T*_^+^ = 0.9	Expected sample size	63.4	50.6
		Probability of rejecting *H*_0_^+^	0.906	0.801
		Probability of detecting heterogeneity (1st stage)	0.840	0.739
3	*p*_*R*_^−^ = 0.9/*p*_*T*_^−^ = 0.9 *p*_*R*_^+^ = 0.9/*p*_*T*_^+^ = 0.9	Expected sample size	85.1	63.5
		Probability of rejecting *H*_0_^−^ and *H*_0_^+^	0.826	0.800
**Case study 2**				
		Maximum sample size	62	39
1A	*p*_*R*_^−^ = 0.3/*p*_*T*_^−^ = 0.9 *p*_*R*_^+^ = 0.3/*p*_*T*_^+^ = 0.9	Expected sample size	34.5	25.7
		Probability of early termination	0.430	0.555
		Probability of rejecting *H*_0_^−^ or *H*_0_^+^	0.083	0.093
1B	*p*_*R*_^−^ = 0.6/*p*_*T*_^−^ = 0.6 *p*_*R*_^+^ = 0.6/*p*_*T*_^+^ = 0.6	Expected sample size	35.1	24.3
		Probability of early termination	0.408	0.627
		Probability of rejecting *H*_0_^−^ or *H*_0_^+^	0.054	0.092
2	*p*_*R*_^−^ = 0.3/*p*_*T*_^−^ = 0.6 *p*_*R*_^+^ = 0.6/*p*_*T*_^+^ = 0.9	Expected sample size	42.4	31.0
		Probability of rejecting *H*_0_^+^	0.905	0.802
		Probability of detecting heterogeneity (1st stage)	0.807	0.799
3	*p*_*R*_^−^ = 0.6/*p*_*T*_^−^ = 0.9 *p*_*R*_^+^ = 0.6/*p*_*T*_^+^ = 0.9	Expected sample size	59.2	37.4
		Probability of rejecting *H*_0_^−^ and *H*_0_^+^	0.821	0.800
**Case study 3**				
		Maximum sample size	79	45
1A	*p*_*R*_^−^ = 0.1/*p*_*T*_^−^ = 0.8 *p*_*R*_^+^ = 0.1/*p*_*T*_^+^ = 0.9	Expected sample size	43.7	31.2
		Probability of early termination	0.511	0.620
		Probability of rejecting *H*_0_^−^ or *H*_0_^+^	0.055	0.086
1B	*p*_*R*_^−^ = 0.4/*p*_*T*_^−^ = 0.6 *p*_*R*_^+^ = 0.4/*p*_*T*_^+^ = 0.6	Expected sample size	44.8	32.0
		Probability of early termination	0.493	0.582
		Probability of rejecting *H*_0_^−^ or *H*_0_^+^	0.083	0.096
2	*p*_*R*_^−^ = 0.1/*p*_*T*_^−^ = 0.6 *p*_*R*_^+^ = 0.4/*p*_*T*_^+^ = 0.9	Expected sample size	45.7	34.9
		Probability of rejecting *H*_0_^+^	0.904	0.802
		Probability of detecting heterogeneity (1st stage)	0.864	0.825
3	*p*_*R*_^−^ = 0.4/*p*_*T*_^−^ = 0.8 *p*_*R*_^+^ = 0.4/*p*_*T*_^+^ = 0.9	Expected sample size	75.8	43.5
		Probability of rejecting *H*_0_^−^ and *H*_0_^+^	0.821	0.801

In all three case studies, the maximum sample size was larger with the parallel BD design with respectively 88, 62 and 79 patients compared with 67, 39 and 45 patients for the SABD.

Scenarios 1A and 1B, the SABD gave the smallest ESS with a maximum of 42.4, 25.7 and 32.0 patients compared to the parallel BD with a maximum of 52.8, 35.1 and 44.8 patients for the three case studies, respectively. The probability of rejecting *H*_0_^−^ or *H*_0_^+^ (i.e. type I error rates *α*_*R*_ and *α*_*T*_) was approximately 10% for each design and case study (except in scenarios 1A and 1B for case study 2 and 3 with the parallel BD, respectively). The PET varied between 41 and 46% for case study 1 and was higher when using the SABD with a minimum of 55.5% and 58.2% compared to the parallel BD with a minimum of 40.8% and 49.3% for the case studies 2 and 3, respectively.

In scenario 2, for each case study, the probability of rejecting *H*_0_^+^ is higher when using the parallel BD (approximately 90%) compared to the SABD (approximately 80%). The probability of detecting heterogeneity at the first stage was at least 80% for each design and case study, except for the SABD in case study 1 (73.9%). The SABD gave the smallest ESS with respectively 50.6, 31.0 and 34.9 patients compared to the parallel BD with 63.4, 42.4 and 45.7 patients for the three case studies.

In scenario 3, the probability of rejecting *H*_0_^−^ and *H*_0_^+^ was approximately 80% for each design and case study. The ESS was lower for the three case studies when using the SABD, with 63.5, 37.4 and 43.5 patients compared to 85.1, 59.2 and 75.8 patients for the parallel BD, respectively.

## Discussion

The stratified adaptive phase II design developed and presented in this paper takes into account the heterogeneity of a population when considering co-primary endpoints. The SABD design based on the Jones et al. [8] and Parashar et al. [10] algorithm, allows to include two pre-defined subgroups and to identify whether the therapeutic benefits one of these subgroups at the end of the first or the second stage. Different hypotheses can be defined between the subgroups and/or co-primary endpoints. We used three case studies to simulate different scenarios and investigate the operating characteristics of the SABD approach. The results demonstrate good statistical performances for the SABD when compared to the parallel BD (one BD for each subgroup). The SABD indeed allows to reduce the number of patients exposed to an insufficiently active or overly toxic treatment (scenarios 1A and 1B). The ESS required to reach an adequate statistical power to draw meaningful conclusions in the unselected population is also lower compared to the parallel BD (scenario 3). The same trend is observed in scenario 2 but the parallel BD yields a higher statistical power to conclude to the feasibility of the treatment in the positive subgroup only (i.e. «go-decision»). If there was heterogeneity between the two subgroups, the probability of detecting it at the first stage was generally at least 80%. To account for multiplicity and obtain an adequate overall type I error rate of 10%, *α*_*T*_ and *α*_*R*_ were set at 5% for each BD. In case study 2 and 3, optimal BD designs were determined using binomial probabilities with *α*_*T*_ and *α*_*R*_ less than 3.5%. This could explain the lower type I error rate observed in scenarios 1A and 1B for the parallel BD, compared to the SABD.

Given that the endpoint was two-dimensional, alternative case studies or scenarios may also be considered. It would, for instance, be interesting to investigate the statistical performance of the SABD design when heterogeneity only affects one dimension.

Similarly to the BD design, the SABD design assumes that the co-primary endpoints are independent. An alternative to the BD design which pre-defines the association between co-primary endpoints has also been developed [15]. Such an extension of the SABD design to correlated endpoints implies, among other things, to consider a bivariate binomial distribution with a correlation between the two co-primary endpoints but also between the two subgroups. This merits further investigation. A simulation study assessing the impact of an erroneous assumption of this pre-defined association however recommends using the BD design. Indeed, incorrectly assuming independence of endpoints only slightly increases the type I and II error rates. This is in contrast to wrongly defining the level of correlation between co-primary endpoints which results in a significant loss of statistical power and an increase in the type I error rate [16]. Future studies will be required to investigate the impact of wrongly assuming independence of co-primary endpoints on the performance of stratified design approaches.

The Jones et al. [8] and Parashar et al. [10] algorithm assumes that there is a hierarchy between subgroups, such that the true response and non-toxicity rate will always be higher in the positive subgroup. This may lead to the results of the positive subgroup having no impact on the outcome of the study if promising results are observed in the negative subgroup in the interim and the final analysis. Indeed, if this hierarchy assumption is incorrect, enrollment of an unselected population may be continued even though promising results are only observed in the negative subgroup at the interim analysis. In this scenario, an additional type I error may occur by declaring a «go-decision» in the unselected population in the case where true response and non-toxicity rates are considered as acceptable in the negative subgroup only (i.e. wrongly reject *H*_0_^+^). Zang & Yuan proposed a reverse approach to address this shortfall [17]. The trial is initially only conducted in the positive subgroup and then in the negative subgroup if promising results are observed in the positive subgroup. An alternative two-stage approach has also been published by Dutton & Holmes [18]. In this approach, futility is first tested in the unselected population and then in the negative or positive subgroup depending on whether or not promising results are observed. The impact of an assumption-based error in relation to hierarchy remains to be evaluated and deserves further investigation.

An optimal SABD design requires a total of 15 parameters to be estimated. A similar approach to the one proposed by Jones et al. [8], which is described in «Expected sample size (ESS) and optimal design» section, was used to reduce the number of parameters that needed to be determined and thus also reduce the computational burden. Further work is required to provide technical solutions and to determine optimal designs over the 15-dimensional parameter space.

## Conclusions

The SABD design allows to independently assess two dimensions through co-primary endpoints in a heterogeneous population without dramatically increasing the sample size. This is particularly useful for geriatric clinical oncology trials as it allows to stratify the population according to a geriatric criterion and to identify a subgroup of interest that has an acceptable and clinically relevant benefit-risk ratio at the end of the first or the second stage. As population heterogeneity is not limited to older populations, the SABD design may also be applicable to other study populations such as children or adolescents and young adults [19]. Children populations are heterogeneous particularly in terms of age, with tolerance of a treatment potentially dependent on these aspects [20]. Our novel SABD approach may also be envisaged for phase II trials of targeted therapies based on a biomarker (positive versus negative) to select the appropriate study population for the subsequent phase III trial.

## Supplementary Information

**Additional file 1:** **Supplementary Table 1.** Stratified adaptive Bryant & Day (SABD) designs with *α*_*R*_ = 0.1, *α*_*T*_ = 0.1, *β* = 0.2 (*ESS*_RiTj_ and PET_RiTj_ correspond to *ESS*(*p*_*Ri*_^-^,*p*_*Tj*_^-^,*p*_*Ri*_^+^,*p*_*Tj*_^+^) and *PET*(*p*_*Ri*_^-^,*p*_*Tj*_^-^,*p*_*Ri*_^+^,*p*_*Tj*_^+^), respectively).

## Data Availability

The data generated and used during this study are available from the corresponding author on reasonable request. The R program implementing the proposed SABD design is available from the corresponding author upon request.

## Abbreviations

BD: Bryant & Day
SABD: Stratified Adaptive Bryant & Day
ESS: Expected Sample Size
PET: Probability of Early Termination
