# Supplementary material for: A stratified adaptive two-stage design with co-primary endpoints for phase II clinical oncology trials
Source: BMC Med Res Methodol. 2022 Oct 26;22:278. doi: 10.1186/s12874-022-01748-w (PMC9608934; doi:10.1186/s12874-022-01748-w)
Supplement: Supplementary file 1 — Additional file 1: Supplementary Table 1. Stratified adaptive Bryant & Day (SABD) designs with αR = 0.1, αT = 0.1, β = 0.2 (ESSRiTj and PETRiTj correspond to ESS(pRi-,pTj-,pRi+,pTj+) and PET(pRi-,pTj-,pRi+,pTj+), respectively). [file 12874_2022_1748_MOESM1_ESM.docx]

Supplementary Table 1 : Stratified adaptive Bryant & Day (SABD) designs with *α_R_* = 0.1, *α_T_* = 0.1, *β* = 0.2 (*ESS*_RiTj_ and PET_RiTj_ correspond to *ESS*(*p_Ri_*^-^,*p_Tj_*^-^,*p_Ri_*^+^,*p_Tj_*^+^) and *PET*(*p_Ri_*^-^,*p_Tj_*^-^,*p_Ri_*^+^,*p_Tj_*^+^), respectively)

| **Hypotheses** | | | | **Parameters** | | | | | | | | **Operating characteristics** | |
| --- | --- | --- | --- | --- | --- | --- | --- | --- | --- | --- | --- | --- | --- |
| *p_R_*_0_ | *p_R_*_1_^-^  *p_R_*_1_^+^ | *p_T_*_0_ | *p_T_*_1_^-^  *p_T_*_1_^+^ | *k*_R1_^-^  *k*_R1_^+^ | *k*_T1_^-^  *k*_T1_^+^ | *N*_1_^-^  *N*_1_^+^ | *k*_Re_^+^  *k*_Te_^+^ | *N*_e_^+^ | *k*_R_^-^  *k*_R_^+^ | *k*_T_^-^  *k*_T_^+^ | *N*^-^  *N*^+^ | max  (*ESS_R_*_0T1_,*ESS_R_*_1T0_) | min  (*PET*_R0T1,_*PET*_R1T0_) |
|  | | | | | | | | | | | | | |
| 0.10 | 0.30  0.30 | 0.50 | 0.70  0.70 | 2  2 | 8  8 | 14  14 | 9  33 | 54 | 9  6 | 31  19 | 50  29 | 56.6 | 0.389 |
| 0.10 | 0.30  0.40 | 0.50 | 0.70  0.70 | 2  3 | 8  11 | 14  18 | 9  32 | 52 | 9  5 | 31  16 | 50  24 | 52.9 | 0.475 |
| 0.10 | 0.30  0.30 | 0.50 | 0.70  0.80 | 2  2 | 8  7 | 14  12 | 6  19 | 29 | 9  4 | 31  12 | 50  17 | 45.2 | 0.403 |
| 0.10 | 0.30  0.40 | 0.50 | 0.70  0.80 | 2  2 | 8  6 | 14  9 | 6  17 | 26 | 9  3 | 31  10 | 50  13 | 40.6 | 0.476 |
|  | | | | | | | | | | | | | |
| 0.30 | 0.50  0.50 | 0.50 | 0.70  0.70 | 7  7 | 11  11 | 19  19 | 23  36 | 60 | 26  19 | 39  28 | 65  45 | 68.8 | 0.481 |
| 0.30 | 0.50  0.60 | 0.50 | 0.70  0.70 | 7  7 | 11  11 | 19  18 | 20  30 | 49 | 26  13 | 39  21 | 65  32 | 60.5 | 0.528 |
| 0.30 | 0.50  0.50 | 0.50 | 0.70  0.80 | 7  7 | 11  11 | 19  18 | 16  25 | 39 | 26  15 | 39  20 | 65  32 | 59.4 | 0.504 |
| 0.30 | 0.50  0.60 | 0.50 | 0.70  0.80 | 7  4 | 11  6 | 19  10 | 10  15 | 22 | 26  9 | 39  12 | 65  16 | 47.8 | 0.453 |
|  | | | | | | | | | | | | | |
| 0.50 | 0.70  0.70 | 0.50 | 0.70  0.70 | 11  11 | 11  11 | 19  19 | 34  34 | 57 | 38  31 | 38  31 | 63  49 | 67.9 | 0.495 |
| 0.50 | 0.70  0.80 | 0.50 | 0.70  0.70 | 11  10 | 11  10 | 19  17 | 25  25 | 40 | 38  20 | 38  21 | 63  31 | 58.2 | 0.485 |
| 0.50 | 0.70  0.70 | 0.50 | 0.70  0.80 | 11  10 | 11  10 | 19  17 | 25  27 | 40 | 38  23 | 38  21 | 63  35 | 59.4 | 0.485 |
| 0.50 | 0.70  0.80 | 0.50 | 0.70  0.80 | 11  5 | 11  5 | 19  8 | 14  14 | 21 | 38  14 | 38  14 | 63  18 | 46.2 | 0.462 |
|  | | | | | | | | | | | | | |
| 0.10 | 0.30  0.30 | 0.60 | 0.80  0.80 | 2  2 | 9  9 | 13  13 | 9  37 | 52 | 9  6 | 35  21 | 49  28 | 52.2 | 0.434 |
| 0.10 | 0.30  0.40 | 0.60 | 0.80  0.80 | 2  3 | 9  12 | 13  17 | 7  30 | 42 | 9  4 | 35  15 | 49  18 | 46.6 | 0.495 |
| 0.10 | 0.30  0.30 | 0.60 | 0.80  0.90 | 2  2 | 9  8 | 13  11 | 5  20 | 26 | 9  5 | 35  14 | 49  18 | 41.6 | 0.463 |
| 0.10 | 0.30  0.40 | 0.60 | 0.80  0.90 | 2  2 | 9  7 | 13  9 | 4  15 | 19 | 9  4 | 35  9 | 49  10 | 36.0 | 0.518 |
|  | | | | | | | | | | | | | |
| 0.30 | 0.50  0.50 | 0.60 | 0.80  0.80 | 7  7 | 13  13 | 19  19 | 19  34 | 48 | 24  18 | 42  29 | 59  41 | 63.6 | 0.474 |
| 0.30 | 0.50  0.60 | 0.60 | 0.80  0.80 | 7  5 | 13  9 | 19  13 | 16  26 | 36 | 24  11 | 42  20 | 59  26 | 53.5 | 0.472 |
| 0.30 | 0.50  0.50 | 0.60 | 0.80  0.90 | 7  6 | 13  11 | 19  15 | 18  32 | 44 | 24  15 | 42  24 | 59  35 | 58.2 | 0.499 |
| 0.30 | 0.50  0.60 | 0.60 | 0.80  0.90 | 7  4 | 13  7 | 19  9 | 10  16 | 21 | 24  8 | 42  13 | 59  16 | 44.8 | 0.512 |
|  | | | | | | | | | | | | | |
| 0.50 | 0.70  0.70 | 0.60 | 0.80  0.80 | 12  12 | 14  14 | 21  21 | 29  33 | 47 | 31  26 | 36  31 | 50  41 | 63.7 | 0.454 |
| 0.50 | 0.70  0.80 | 0.60 | 0.80  0.80 | 12  8 | 14  9 | 21  13 | 23  26 | 36 | 31  18 | 36  22 | 50  28 | 53.6 | 0.443 |
| 0.50 | 0.70  0.70 | 0.60 | 0.80  0.90 | 12  10 | 14  12 | 21  17 | 24  28 | 38 | 31  22 | 36  24 | 50  33 | 56.8 | 0.469 |
| 0.50 | 0.70  0.80 | 0.60 | 0.80  0.90 | 12  6 | 14  7 | 21  9 | 13  15 | 19 | 31  12 | 36  13 | 50  16 | 43.2 | 0.518 |
